# Inside-out Technique using the Suture Gripping Technique Enabling Withdrawal of Suture from Outside the Joint with a Suture Shuttle for Meniscal Suturing

**DOI:** 10.1016/j.eats.2025.103823

**Published:** 2025-08-29

**Authors:** Ryo Sasaki, Hitomi Tsunashima, Yoshihiro Baba, Taichi Nishimura, Teppei Hayashi, Kazuya Kaneda, Masaki Nagashima, Hideo Morioka

**Affiliations:** a Department of Orthopaedic Surgery, National Hospital Organization, Tokyo Medical Center, Tokyo, Japan; b Department of Orthopaedic Surgery, Keio University School of Medicine, Tokyo, Japan; c Department of Orthopaedic Surgery, International University of Health and Welfare Mita Hospital, Tokyo, Japan

## Abstract

Various meniscal repair techniques, including all-inside, outside-in, and inside-out methods, have been developed; however, they require complex maneuvers with each method, and there are limitations to the suture material. This report presents an arthroscopic inside-out technique for meniscal repair using a suture-gripping method with a suture shuttle, which allows insertion of the needle into the meniscus from inside the knee and withdrawal of the suture from outside the knee. This technique allows free gripping of the suture, making it possible for the suture to pass more accurately than with the standard outside-in technique, and enables a more flexible meniscal repair than that achieved with the standard inside-out technique. This technique overcomes the limitations of other techniques and offers flexibility in suture materials and methods. Furthermore, this technique is effective for suturing the anterior portion of the meniscus because the suture shuttle is more compact and flexible than the devices used in other techniques. This article aims to describe the benefits of this technique for suturing meniscal tears in a more flexible, straightforward, and minimally invasive manner.

The meniscus is an integral component of the knee joint and is vital for knee function. It has functions in joint stability, shock absorption, load transmission, proprioception, and articular cartilage nutrition.^1^ Therefore, repair and preservation of the meniscal tissue are ideal and represent critical issues.

Various meniscal repair methods, including all-inside, inside-out, and outside-in techniques, have been described in the literature.^1^ The outside-in and all-inside techniques are commonly used for suturing the anterior portion of the meniscus.^2^^,^^3^ However, these techniques have some limitations. The outside-in technique involves complex intra-articular maneuvers for suture winding and insertion of the needle from the outside to the optimal meniscus position, thus requiring extensive experience and additional tools. Furthermore, sensing the relative positions of the spinal needle and capsule is difficult, especially for inexperienced surgeons, and this process is challenging if the damaged meniscus is unstable.^4^, ^5^, ^6^, ^7^ Additionally, there are limitations to the suture material because only sutures that fit the diameter of the hollow needle used in the outside-in technique can be used. In the all-inside technique, the knot remains inside the joint, raising concerns about cartilage damage caused by the knot.^3^

This technical note describes an inside-out technique using a suture-gripping method with a suture shuttle. This method involves accurately inserting the needle of the suture shuttle into the meniscus from inside the joint while flexibly withdrawing the suture from the outside. With this technique, the suture is much easier to control to achieve the optimal position compared with the standard outside-in technique; additionally, there are no restrictions on the suturing methods or suture materials. Furthermore, this method allows suturing of the anterior portion of the meniscus (Video 1).

## Surgical Technique

### Demonstration of the Suture-Gripping Technique Using a Suture Shuttle

**Fig 1:**
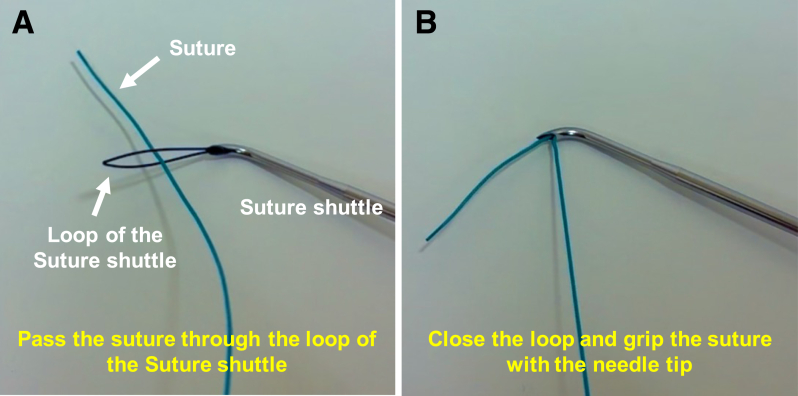
Suture-gripping technique with a suture shuttle. First, the loop of the suture shuttle is expanded, and the suture is passed through the loop (A). Then, the loop is closed, and the suture is gripped with the needle tip (B).

First, the loop of the suture shuttle is expanded, and the suture is passed through the loop (Fig 1A). Then, the loop is closed, and the suture is gripped with the needle tip (Fig 1B). This technique is performed outside the joint when suturing the meniscus.

### Patient Preparation

**Fig 2:**
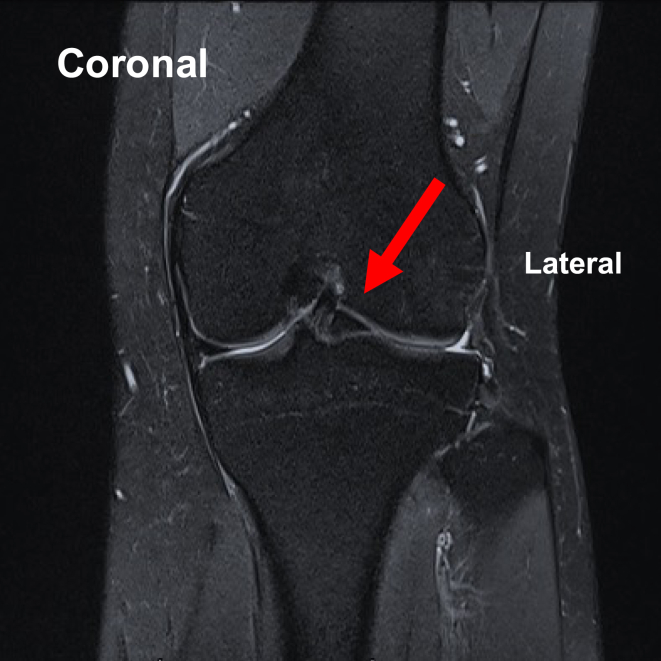
Preoperative coronal magnetic resonance imaging showing a bucket-handle tear of the lateral meniscus (arrow) in the left knee.

Pearls and pitfalls are presented in Table 1. For preoperative preparation, magnetic resonance imaging is performed to evaluate the meniscal tears (Fig 2).

**Table 1:** Pearls and Pitfalls

	Pearls	Pitfalls
Indications	All meniscal tears that can be repaired using an arthroscope	It is important to confirm that the meniscal tear is suitable for arthroscopic repair prior to proceeding.
Suture-gripping technique with SS	Fully expand the loop of the SS prior to passing the suture through it (Fig 1A). Close the loop and securely grip the suture with the needle tip (Fig 1B).	Incomplete loop expansion can lead to failure to pass the suture through the loop. Inadequate gripping may cause the suture to slip or become lost during withdrawal.
Preparation	Perform standard knee arthroscopy using the anterolateral and anteromedial portals to ensure adequate visualization.	Inadequate visualization can lead to insufficient evaluation of the meniscus.
First inside-out insertion of SS (empty suture)	Visualize the anterior portion of the lateral meniscus from the anterolateral portal in the figure-of-4 position. Insert the SS (empty suture) into the meniscotibial capsule and extra-articular space from the anteromedial portal (Fig 3).	Inadequate visualization can result in failure to achieve meniscal suturing. If the skin is too thick for needle penetration, the surgeon should consider making a skin incision prior to insertion.
Gripping of suture outside joint and withdrawal into joint space	Grip the suture in the extra-articular space using the suture-gripping technique with the SS (Fig 4). Withdraw the SS (gripping suture) into the joint space carefully (Fig 5A).	Improper withdrawal may cause the suture to slip out of the loop.
Second inside-out insertion of SS (gripping suture)	Insert the SS (gripping suture) into the meniscus and extra-articular space (Fig 5B). Expand the loop of the SS and withdraw the suture from the loop. Withdraw the SS (empty suture) into the joint space (Fig 5C).	Failure to maintain adequate distance from the first insertion site may result in meniscal cutout. Incomplete release of the suture may lead to entanglement or inadvertent re-withdrawal of the suture into the joint space.
Tensioning and knot tying	After the desired sutures are completed (Fig 6A), make a skin incision near the exit point of the suture and pass the thread subcutaneously to this skin incision (Fig 6B). Visualize the anterior portion of the lateral meniscus from the anteromedial portal, adjust to the appropriate tension, and tie the suture (Fig 6C).	Improper placement of the skin incision or inadequate subcutaneous dissection may result in suture loosening after knot tying. Over- or under-tensioning the suture can result in failed healing or retear.

SS, suture shuttle.

Under general anesthesia, the patient is placed in the supine position on the operating table with a standard leg holder, allowing full range of motion (ROM). Standard knee arthroscopy is performed through the anterolateral and anteromedial portals. Complete diagnostic arthroscopy is performed initially. In this case, the lateral meniscus has a chronic bucket-handle tear; therefore, the displaced lateral meniscus is reduced, and the posterior body of the meniscus and the posterior joint capsule are sutured using the all-inside technique (FAST-FIX FLEX; Smith & Nephew Endoscopy, Andover, MA), with the knee in the figure-of-4 position. Next, the longitudinal tear of the anterior body of the lateral meniscus is sutured using the inside-out suture-gripping technique with a suture shuttle.

### First Inside-Out Insertion of Suture Shuttle (Empty Suture)

**Fig 3:**
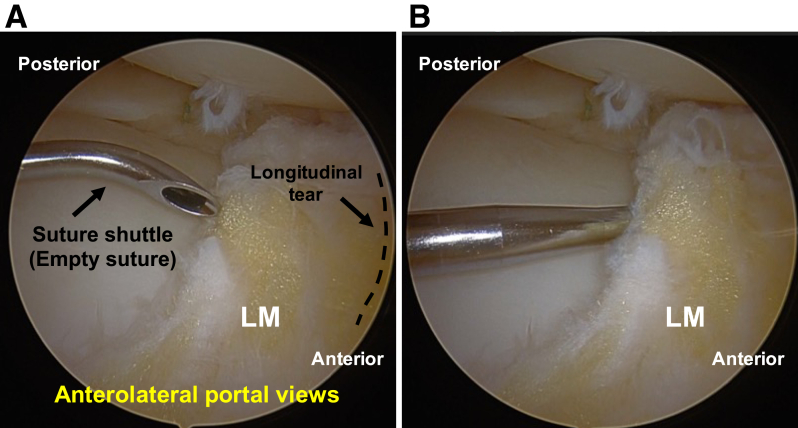
Diagnosis of meniscus injury and first inside-out insertion of the suture shuttle (empty suture) in the left knee, positioned supine in the figure-of-4 position, viewed from the anterolateral portal. The patient is placed in the supine position on the operating table with a standard leg holder, allowing full range of motion. (A) The diagnosis of a longitudinal tear (dashed line) of the anterior body of the lateral meniscus (LM) is made. (B) The needle of the suture shuttle is inserted with an empty suture into the meniscotibial capsule and extra-articular space from the anteromedial portal.

First, the anterior portion of the lateral meniscus is visualized from the anterolateral portal in the figure-of-4 position. Next, the needle of the suture shuttle (ACCU-PASS Suture Shuttle 70° Upbend; Smith & Nephew Endoscopy) is inserted with an empty suture into the meniscotibial capsule and extra-articular space from the anteromedial portal (Fig 3).

### Gripping of Suture Outside Joint and Withdrawal Into Joint Space

**Fig 4:**
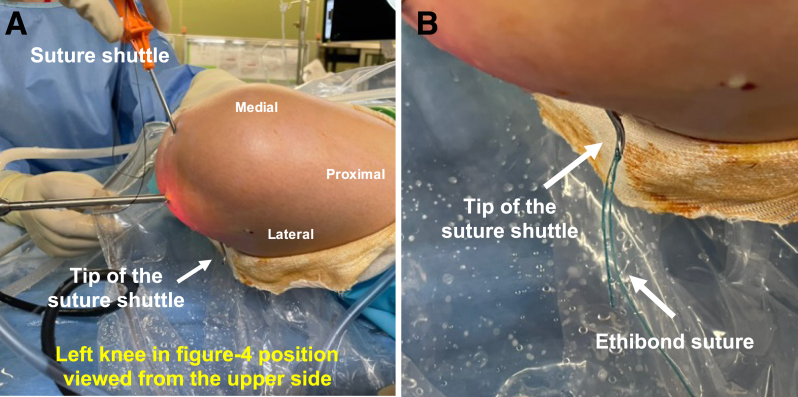
Gripping of the suture outside the joint. (A, B) The No. 2 Ethibond suture is gripped in the extra-articular space using the suture-gripping technique with the suture shuttle, in the left knee in the figure-of-4 position.

**Fig 5:**
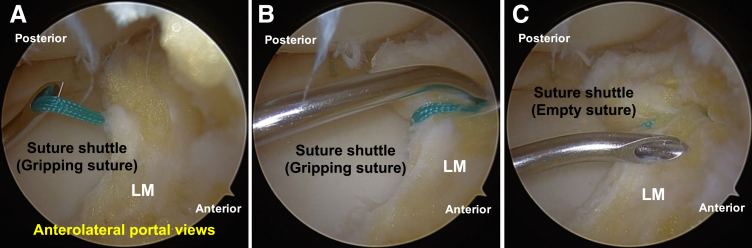
Withdrawal of the suture shuttle into the joint space and second inside-out insertion of the suture shuttle (gripping suture) in the left knee, positioned supine in the figure-of-4 position, viewed from the anterolateral portal. (A) The suture shuttle is withdrawn by gripping the No. 2 Ethibond suture into the joint space. (B) The suture shuttle is inserted, gripping the No. 2 Ethibond suture, into the lateral meniscus (LM) and extra-articular space. (C) The loop of the suture shuttle is expanded, and the suture is withdrawn from the loop. Finally, the suture shuttle, which is now empty, is withdrawn into the joint space.

The suture (No. 2 Ethibond; Ethicon, Raritan, NJ) is gripped in the extra-articular space using the suture-gripping technique with the suture shuttle (Fig 4). Then, the suture shuttle is withdrawn, while gripping the No. 2 Ethibond suture, into the joint space (Fig 5A).

### Second Inside-Out Insertion of Suture Shuttle (Gripping Suture)

The suture shuttle is inserted, while gripping the No. 2 Ethibond suture, into the meniscus and extra-articular space (Fig 5B). Then, the loop of the suture shuttle is expanded, and the suture is withdrawn from the loop. Finally, the suture shuttle, which is now empty, is withdrawn into the joint space (Fig 5C).

### Tensioning and Knot Tying

**Fig 6:**
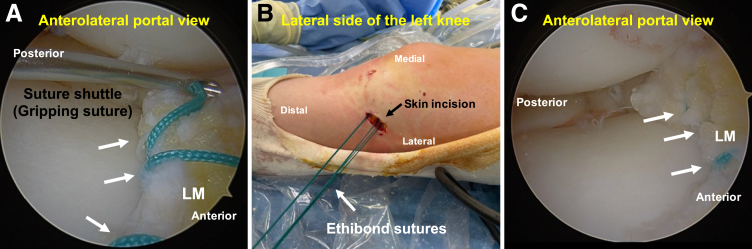
Tensioning and knot tying in the left knee, positioned supine in the figure-of-4 position, viewed from the anterolateral portal. The suturing process is repeated until the desired sutures are completed. (A) In this case, 3 sutures are placed in the anterior portion of the lateral meniscus (LM) using No. 2 Ethibond suture (arrows). (B) A skin incision is made near the suture exit point, and the thread is passed subcutaneously through this incision. (C) With the knee in the figure-of-4 position, the anterior portion of the lateral meniscus is visualized from the anterolateral portal, the tension is adjusted to the appropriate level, and the No. 2 Ethibond sutures (arrows) are tied.

The aforementioned suturing process is repeated until the desired sutures are completed (Fig 6A). A skin incision is made near the suture exit point, and the thread is passed subcutaneously through this incision (Fig 6B). An appropriately placed skin incision and adequate subcutaneous dissection are necessary to adequately expose the site where the suture penetrates the joint capsule; otherwise, loosening of the suture after knot tying may occur. The anterior portion of the lateral meniscus is visualized from the anteromedial portal, the tension is adjusted to the appropriate level, and the No. 2 Ethibond suture is tied (Fig 6C).

### Postoperative Management

**Fig 7:**
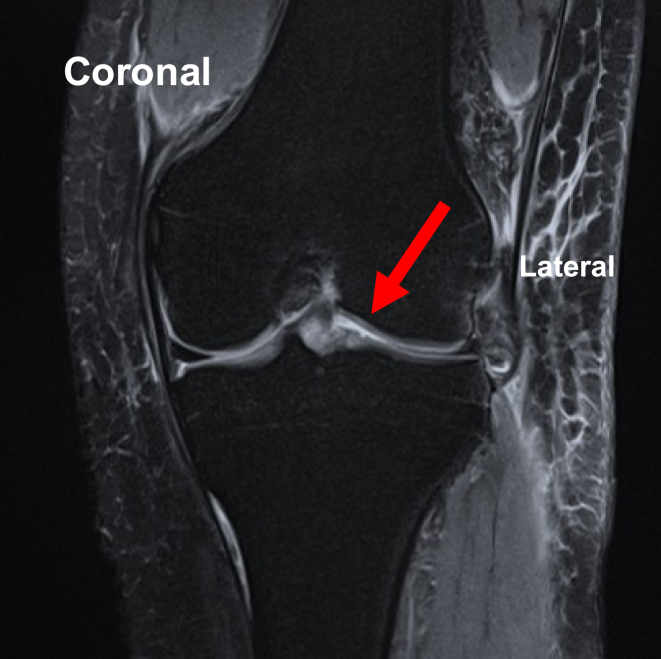
Postoperative coronal magnetic resonance imaging showing the reduction of the lateral meniscus tear (arrow) in the left knee.

The knee is immobilized in full extension in a brace for 1 week after the operation, during which non–weight-bearing is implemented. From 1 week postoperatively, ROM training with a limit of less than 90° is initiated, and from 4 weeks postoperatively, ROM training without range restrictions begins. Full weight-bearing is allowed at 1 week postoperatively with a hinged brace. Postoperative magnetic resonance imaging shows anatomic reduction (Fig 7).

## Discussion

With advancements in arthroscopic equipment, various techniques for meniscal repair have been reported. However, when performing the outside-in technique—especially for unstable tears in the anterior portion of the meniscus—complex maneuvers and advanced skills are required,^2^^,^^3^ and there are limitations to the suture material. In this article, we propose a method that uses a suture-gripping technique with a suture shuttle, allowing insertion of the needle into the optimal position of the meniscus from inside the joint while guiding the suture from outside, which makes the suturing technique particularly straightforward.

The key points of this technique are presented in Table 2. First, this inside-out technique uses a suture shuttle, which allows free gripping of the suture. Our technique facilitates accurate insertion of the needle into the optimal position of the meniscus compared with that achieved by the outside-in technique, and the suture can be flexibly pulled from outside the knee joint into the joint using the suture-gripping technique with a suture shuttle. Second, this method allows more accurate, flexible, and easy meniscal suturing. Some devices used in the inside-out technique can also be used to suture the meniscus, but they are bulkier than a suture shuttle, resulting in reduced operability. Depending on suture location, it is necessary to change the type and degree of bending of the device. In particular, when suturing the anterior portion of the meniscus using the inside-out technique, the device should have a considerable bend; hence, a greater force may be required to pass the needle through the device. Considering the disadvantages of the inside-out technique, the suture shuttle is compact and can approach various meniscal locations with a single device, making this technique more flexible and easy.

**Table 2:** Key Points, Advantages, and Limitations of Procedure

Key points
This method is an inside-out technique using an SS.
This technique allows for gripping of the suture freely.
This technique allows insertion of the needle accurately into the meniscus from inside the knee and withdrawal of the suture flexibly from outside the knee.
Using this method allows the surgeon to perform meniscus suturing more accurately, flexibly, and easily.
Advantages
This technique does not impose restrictions on the suturing method, including continuous suturing, as well as the type of suture material and the number of sutures.
This technique can be used for suturing the anterior portion of the meniscus.
If the SS cannot reach the meniscus from the standard anteromedial or anterolateral portals, the suture can be passed through the far-medial or far-lateral portal.
A suturing knot is created outside the joint capsule.
Limitations
If creating medial or far-lateral portals does not allow the SS to reach the meniscus, this method may not be applicable.
In patients with obesity in whom the SS cannot reach the outside of the joint, it is necessary to create a skin incision and perform subcutaneous dissection before inserting the needle of the SS.
Risks
As with standard outside-in and inside-out techniques, there are risks of soft-tissue damage from the knot of the suture outside the joint capsule.

SS, suture shuttle.

The advantages of this technique are as follows: First, it does not impose restrictions on the suturing method, including continuous suturing, as well as the type of suture material and the number of sutures. Various methods of continuous suturing have been reported to achieve highly secure meniscus suturing,^8^, ^9^, ^10^ all of which can be performed using this technique. Second, this technique can be used to suture the anterior portion of the meniscus. If the suture shuttle cannot reach the meniscus from the standard anteromedial or anterolateral portals, suturing is facilitated by creating and passing the suture through the far-medial or far-lateral portal. Third, a suturing knot is created outside the joint capsule, eliminating concerns about joint surface damage caused by an intra-articular knot, as observed in the all-inside technique.

However, this technique has some limitations that must be acknowledged. This method may not be applicable to areas of the meniscus that are unreachable by the suture shuttle, even with the creation of far-medial or far-lateral portals. Additionally, in patients with obesity in whom the suture shuttle cannot reach the outside of the joint, this technique is expected to be difficult. In such cases, it is necessary to create a skin incision and perform subcutaneous dissection before inserting the needle of the suture shuttle outside the joint.

In this article, we describe the inside-out technique using the suture-gripping method with a suture shuttle. This is considered a useful surgical technique owing to its greater flexibility, fewer restrictions, relative ease of use, and safety.

## Disclosures

All authors (R.S., H.T., Y.B., T.N., T.H., K.K., M.N., H.M.) declare that they have no known competing financial interests or personal relationships that could have appeared to influence the work reported in this paper.

## Supplementary Data

Video 1 Inside-out technique using suture-gripping method in the left knee, with the patient positioned supine in the figure-of-4 position, viewed from the anterolateral portal.. Various meniscal repair methods, including all-inside, inside-out, and outside-in techniques, have been developed; however, these involve complex maneuvers, and there are limitations to the suture material. One of the key points of this technique is the use of the suture-gripping method with a suture shuttle, which allows insertion of the needle into the meniscus from inside the knee and withdrawal of the suture from outside the knee. This technique is effective for suturing the anterior portion of the meniscus because the suture shuttle is more compact and flexible than the devices used in other techniques. This technique offers high accuracy, flexibility, and ease of suturing. First, we demonstrate the suture-gripping technique with the suture shuttle. The loop of the suture shuttle is expanded, and the suture is passed through the loop. Then, the loop is closed, and the suture is gripped with the needle tip. This technique is performed outside the joint during meniscal suturing. The patient is a 28-year-old woman who injured her left knee several months before presentation. Magnetic resonance imaging shows a bucket-handle tear of the lateral meniscus in the left knee. Under general anesthesia, the patient is placed in the supine position on the operating table with a standard leg holder, allowing full range of motion. Initially, standard arthroscopy is performed from the anterolateral portal. In this case, the lateral meniscus has a chronic bucket-handle tear; hence, the displaced lateral meniscus is reduced, and the posterior body of the meniscus and the posterior joint capsule are sutured using the all-inside technique, with the knee in the figure-of-4 position. In the anteromedial and anterolateral portal view, with the knee in the figure-of-4 position, a longitudinal tear is observed on the lateral meniscus, and the meniscus is unstable. We suture this tear in an inside-out manner using the suture-gripping technique with a suture shuttle. First, in the anterolateral portal view, with the knee in the figure-of-4 position, the needle of the suture shuttle is inserted with an empty suture into the meniscotibial capsule and extra-articular space from the anteromedial portal. Next, a No. 2 Ethibond suture is placed in the extra-articular space using the suture-gripping technique with the suture shuttle. Then, the suture shuttle is withdrawn, gripping the No. 2 Ethibond suture, into the joint space. Next, the suture shuttle is inserted, gripping the No. 2 Ethibond suture into the meniscus and extra-articular space. The suture shuttle loop is then expanded, and the suture is withdrawn from the loop. Finally, the suture shuttle is withdrawn and emptied into the joint space. After completion of the desired suture, a skin incision is made near the exit point of the suture, and the thread is passed subcutaneously to this incision. With the knee in the figure-of-4 position, the anterior portion of the lateral meniscus is visualized from the anteromedial portal, appropriate tension is achieved, and the No. 2 Ethibond suture is tied.
